# Supplementary figures and images for: The secondary messenger ppGpp interferes with cAMP-CRP regulon by promoting CRP acetylation in Escherichia coli
Source: PLoS One. 2021 Oct 27;16(10):e0259067. doi: 10.1371/journal.pone.0259067 (PMC8550359; doi:10.1371/journal.pone.0259067)

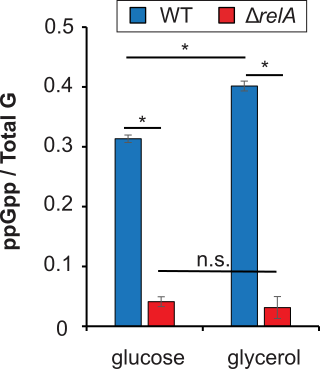

Supplement: S1 Fig — The amount of ppGpp was measured relative to total amount of G (pppGpp + ppGpp + GTP) by TLC in MG1655 and ΔrelA strains grown in MOPS media with 0.2% glucose or 0.4% glycerol. Error bars represent SD of two biological replicates. Statistical significance was measured with T-student test (*p-value < 0.05, n.s. p-value > 0.05). (TIFF) [file pone.0259067.s001.tiff]

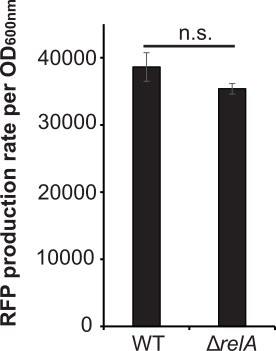

Supplement: S2 Fig — MG1655 (WT) and CF18005 (ΔrelA) harboring the plasmid pBbA5k were grown in M9 minimal media supplemented with glycerol 0.4% and IPTG 1 mM. Finally, fluorescence emitted by RFP (Red Fluorescent Protein) was measured. The amount of fluorescence was plotted against the OD600, giving a linear correlation where the slope is the RFP production rate per OD600. Error bars represent SD. Statistical significance was measured with T-student test (n.s. p-value > 0.05). (TIFF) [file pone.0259067.s002.tiff]

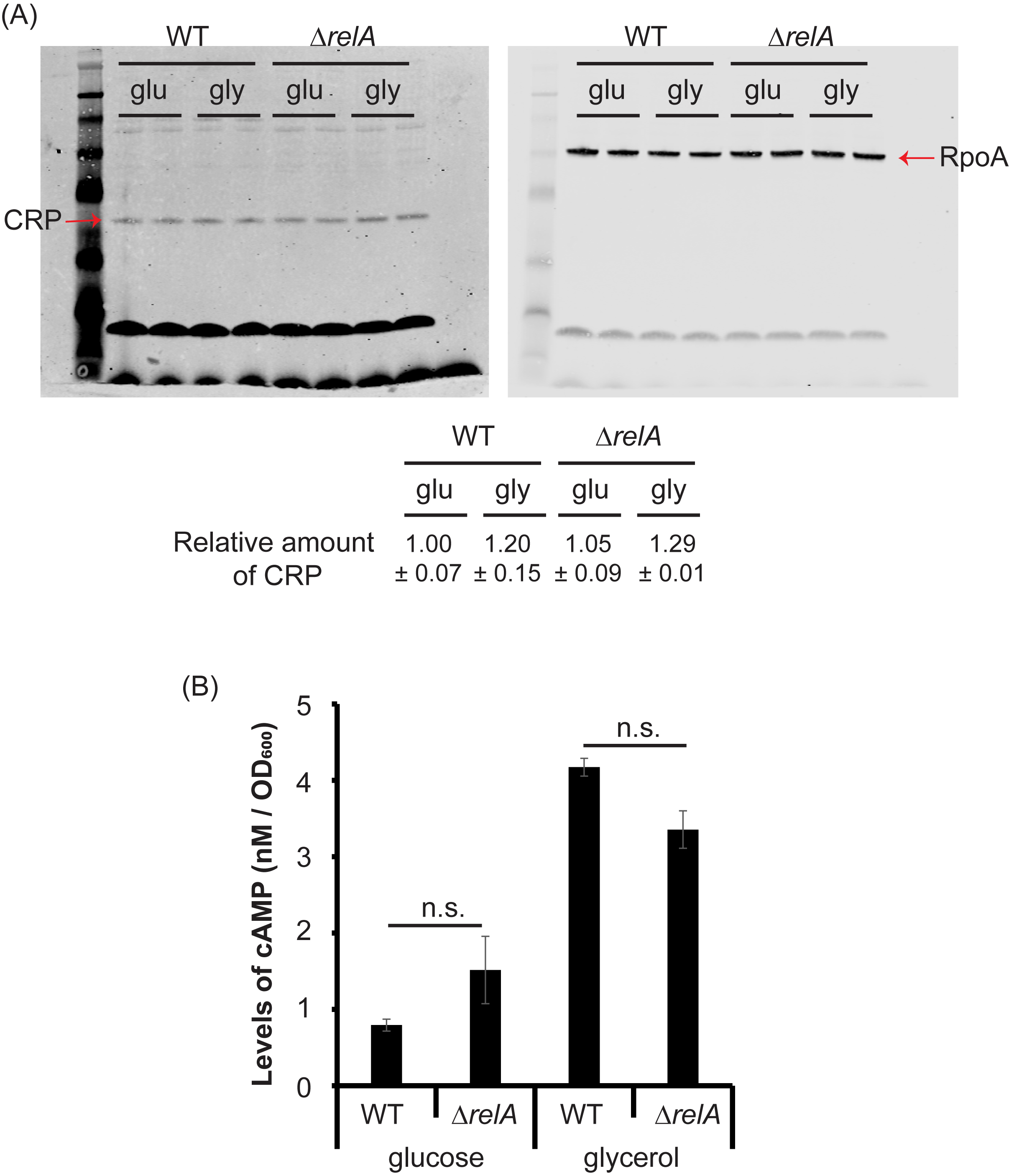

Supplement: S3 Fig — (A) MG1655 (WT) and CF18005 (ΔrelA) were grown in M9 minimal media supplemented with either glucose 0.2% or glycerol 0.4% up to exponential phase (OD600 0.1) and protein levels of CRP and RpoA were measured by Western blot. Means and standard deviation are shown of CRP amounts normalized to the amounts of RpoA (loading control) relative to the values from WT in glucose. (B) Measurements of cAMP of cells grown as in panel (A). Error bars show standard deviation of 2 biological samples. Statistical significance was measured with T-student test (n.s. p-value > 0.05). (TIF) [file pone.0259067.s003.tif]

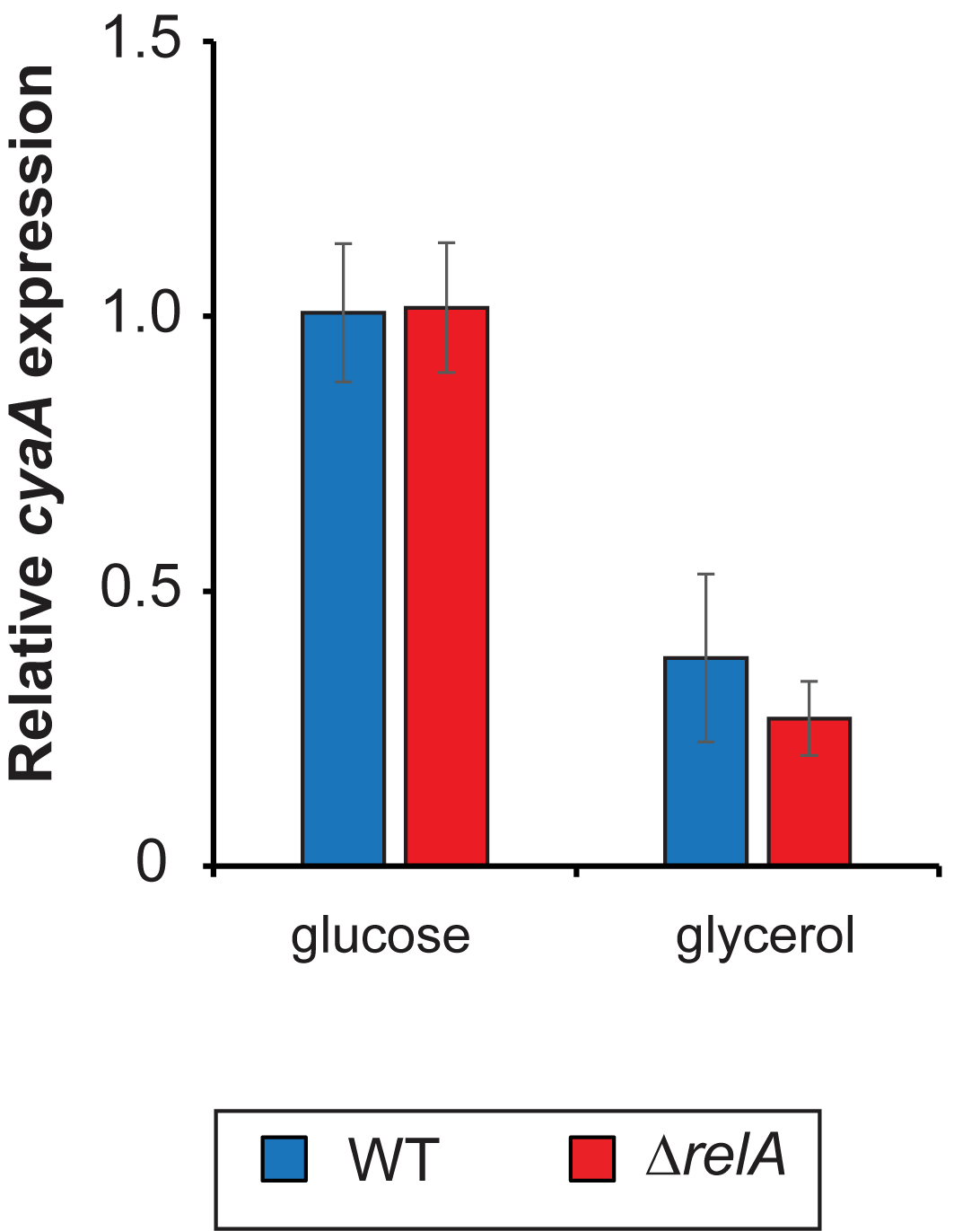

Supplement: S4 Fig — Expression levels are normalized to the expression in WT grown in glucose. Error bars show standard deviation of 2 biological samples and 3 technical replicates. Statistical significance was measured with T-student test and no difference was observed between WT and ΔrelA (p-value > 0.05). (TIF) [file pone.0259067.s004.tif]

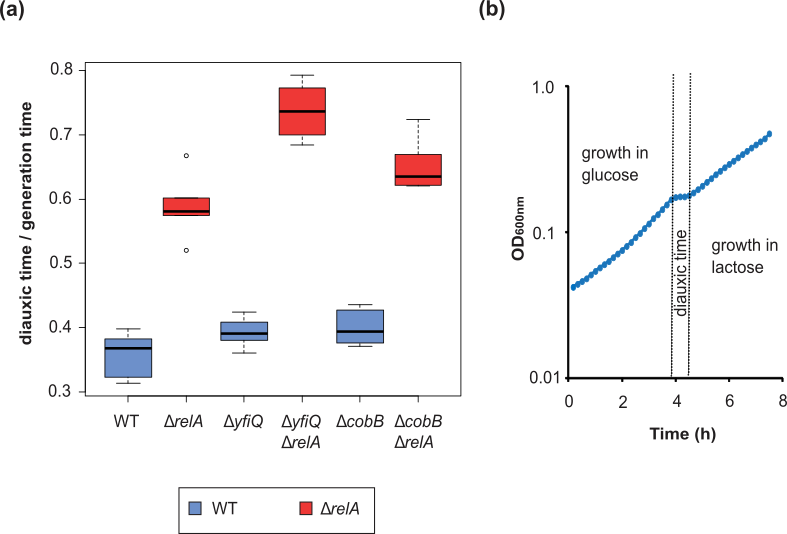

Supplement: S5 Fig — (a) The strain MG1655 (wt) and ΔrelA, together with their isogenic ΔyfiQ and ΔcobB mutants were grown in M9 with 0.025% glucose and 0.4% lactose for 12 h and OD600 measured every 10 min. Ratios of diauxic time normalized to generation times of three independent experiments with duplicate wells (six values) were plotted as box plots. Bottom and top of the colored box represent first and third quartiles, and the band inside the box is the median. Whiskers represent minimum and maximum data, while circles are outliers (single points). (b) Typical diauxic growth curve from WT strain. (TIFF) [file pone.0259067.s005.tiff]

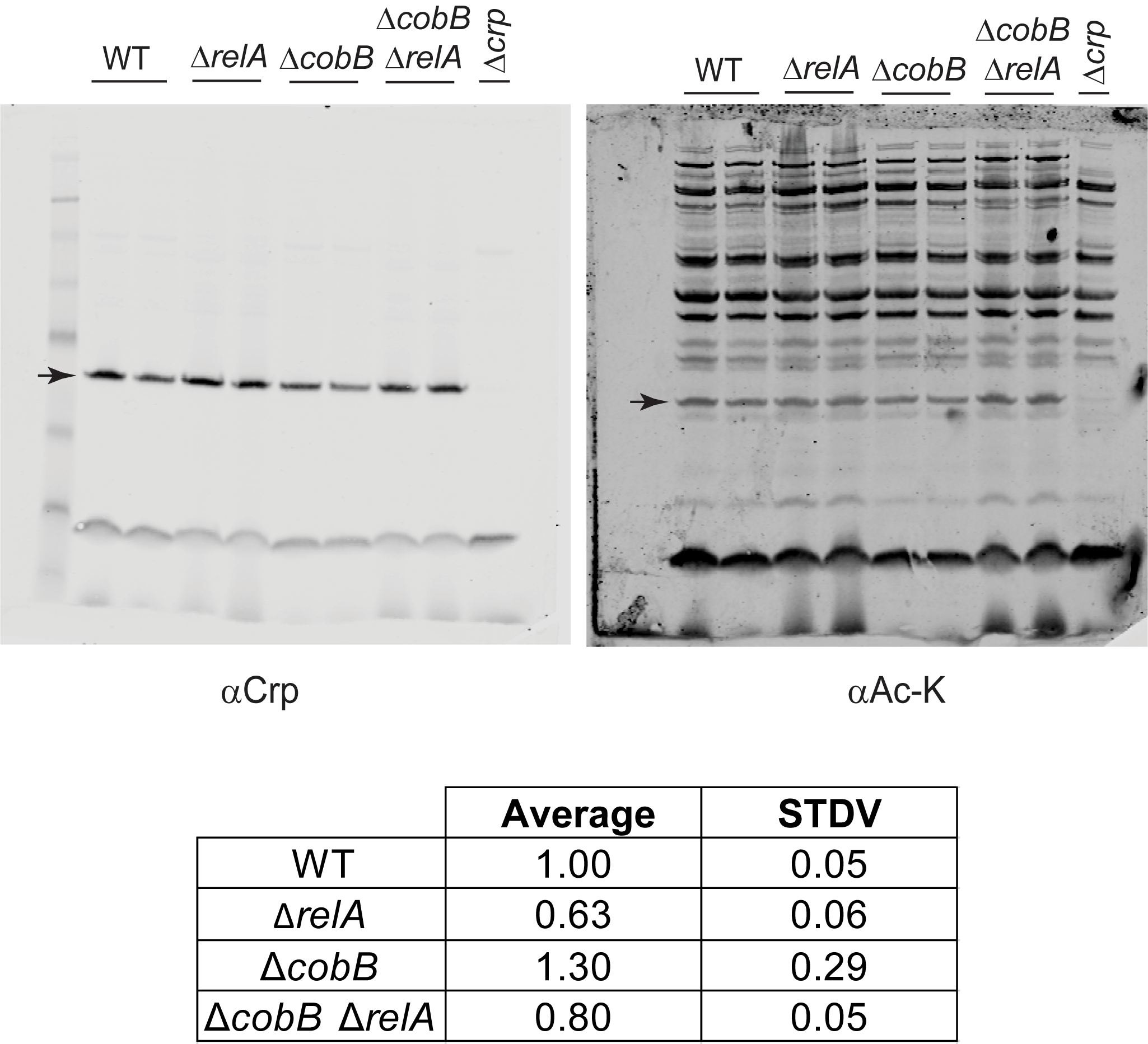

Supplement: S6 Fig — The amount of acylated CRP was measured by Western blot from total extracts of WT and ΔrelA strains together with their isogenic ΔcobB mutants, were grown in M9 minimal media supplemented with glycerol 0.4% up to OD600 of 0.1. To ensure a proper identification of the CRP protein, a Δcrp mutant was also added. The amount of acetylated protein (detected by Western blot using antibody specific for acetylated lysines, Ac-K) was normalized to the amount of CRP and presented relative to the WT strain. STDV = standard deviation. (TIF) [file pone.0259067.s006.tif]

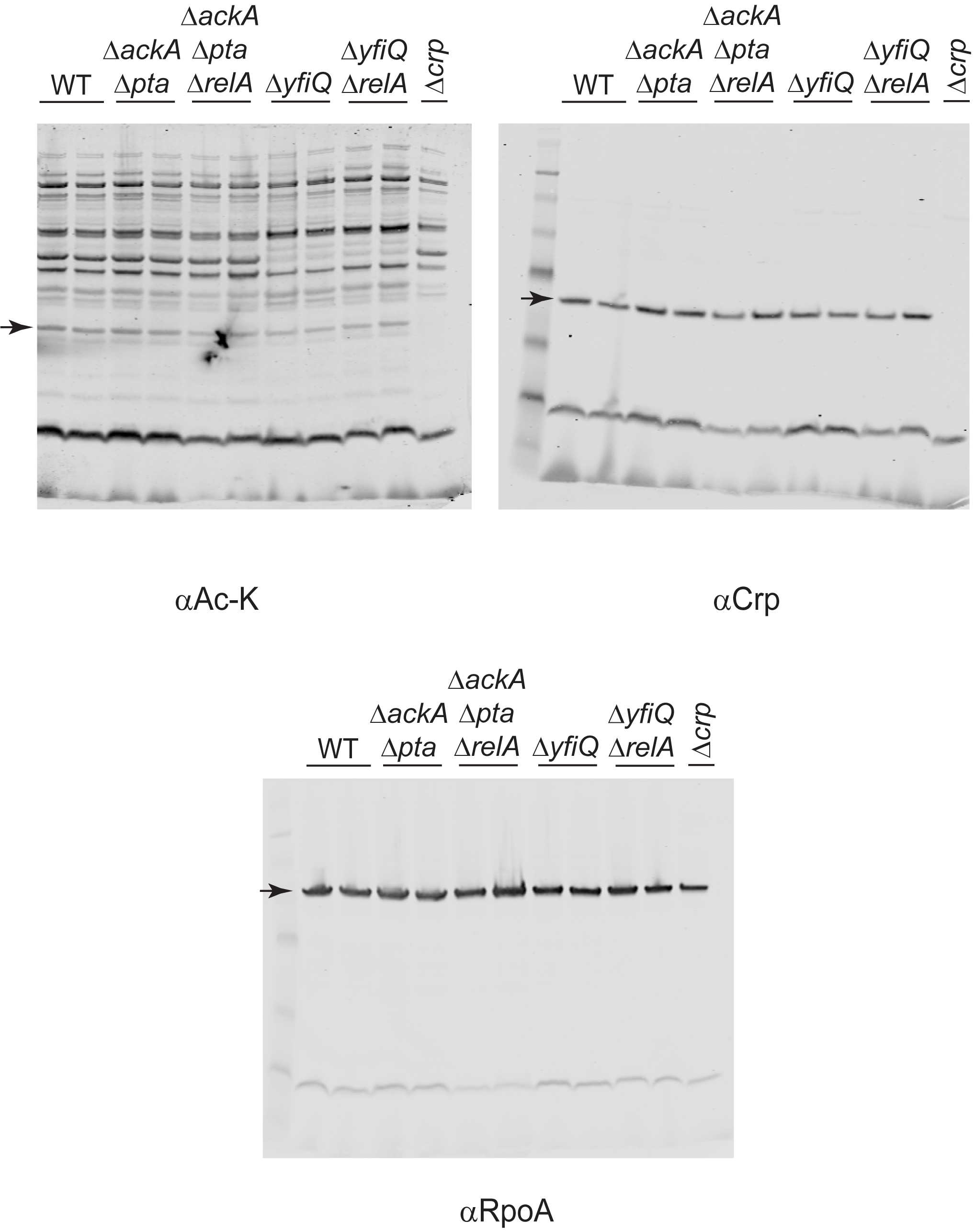

Supplement: S7 Fig — The amount of acylated CRP was measured by Western blot from total extracts of the WT strain, together with the ΔackA-pta, ΔackA-pta ΔrelA, ΔyfiQ and ΔyfiQ ΔrelA strains. Cells were grown in M9 minimal media supplemented with glycerol 0.4% up to exponential phase (OD600 0.1). To ensure a proper identification of the CRP protein, a Δcrp mutant was also added. The amount of acetylated protein (detected by Western blot using antibody specific for acetylated lysines, Ac-K) was normalized to the amount of CRP and presented relative to the WT strain. The CRP amounts are normalized to the amounts of RpoA (loading control). (TIF) [file pone.0259067.s007.tif]
