# Supplementary material for: The secondary messenger ppGpp interferes with cAMP-CRP regulon by promoting CRP acetylation in Escherichia coli
Source: PLoS One. 2021 Oct 27;16(10):e0259067. doi: 10.1371/journal.pone.0259067 (PMC8550359; doi:10.1371/journal.pone.0259067)
Supplement: S1 Table — (PDF) [file pone.0259067.s008.pdf]

S1 Table: Bacterial strains and plasmids used in this study.

Strains		
**Name**	**Properties**	**Origin**
MG1655	F-, *ilvG*, *rph*1	[1]
CF18005	MG1655 Δ*relA*256 (CF12510)	[2]
CF18565	MG1655 Δ*ackA*Δ*ptazej223*::Tn10	[3]
CF18531	CF18005Δ*ackA*Δ*ptazej223*::Tn10	This study
CF18572	MG1655 *yfiQ*::Km	This study
LFC1501	CF18005 *yfiQ*::Km	This study
CF18566	MG1655 *cobB*::Km	This study
CF18582	CF18005 *cobB*::Km	This study
CF6271	MG1655 crp5::Cm	Cashel Lab
BW16470	DE3(*lac*)X74 *phoR68*(Oc) Δ(*creABCD*)114/104::Tn5-112 Δ(*pta-ackA-hisQ-hisP*) zej223::Tn10	[4]
**Plasmids**		
**Name**	**Properties**	**Origin**
pBbA5k	Km^R, p15A origin, RFP under a lacUV5 promoter	[6]

References

1. Jin, D.J.; Gross, C.A. Mapping and sequencing of mutations in the Escherichia colirpoB gene that lead to rifampicin resistance. *J. Mol. Biol.***1988**, *202*, 45–58.

2. Potrykus, K.; Murphy, H.; Philippe, N.; Cashel, M. ppGpp is the major source of growth rate control in E. coli. *Environ. Microbiol.***2011**, *13*, 563–75.

3. Fernández-Coll, L.; Cashel, M. Contributions of SpoT Hydrolase, SpoT Synthetase, and RelA Synthetase to Carbon Source Diauxic Growth Transitions in Escherichia coli. *Front. Microbiol.***2018**, *9*, 1802.

4. Wanner, B.L.; Wilmes-Riesenberg, M.R. Involvement of phosphotransacetylase, acetate kinase, and acetyl phosphate synthesis in control of the phosphate regulon in Escherichia coli. *J. Bacteriol.***1992**, *174*, 2124–2130.

5. Aiba, H.; Fujimoto, S.; Ozaki, N. Molecular cloning and nucleotide sequencing of the gene for E. coli cAMP receptor protein. *Nucleic Acids Res.***1982**, *10*, 1345–61.

6. Anderson, J.C.; Dueber, J.E.; Leguia, M.; Wu, G.C.; Goler, J.A.; Arkin, A.P.; Keasling, J.D. BglBricks: A flexible standard for biological part assembly. *J. Biol. Eng.***2010**, *4*.
