# Supplementary material for: The secondary messenger ppGpp interferes with cAMP-CRP regulon by promoting CRP acetylation in Escherichia coli
Source: PLoS One. 2021 Oct 27;16(10):e0259067. doi: 10.1371/journal.pone.0259067 (PMC8550359; doi:10.1371/journal.pone.0259067)
Supplement: S2 Table — (PDF) [file pone.0259067.s009.pdf]

S2 Table: List of primers used in this report.

Name	Sequence (from 5′ → 3′)
pta1	AACTGAACGCACCGGTTGAT
pta2	GAAGAGTCGTCGAAAATCTC
ackA1	CAAACTGCTGACCAAAGAGT
ackA2	GCGGTAGTTGTCTTCAACAT
cobB1	GCGCAGGTAATACCAATGTG
cobB2	TAACGTCTCCTGTCCAGTCG
yfiQ1	CCTGTTGGTGCAAAGTATGG
yfiQ2	CTTCACCCAGCATGATCAAC
cyaA1	CTGCTGGCGAAAGATATCAA
cyaA2	GCAGCGACGTACTAAATCCA
spcA1	GCAGAAGAGCAAGGTGTTAC
spcA2	GACGGTTTTGGTGGGTAAAT
