# Supplementary material for: The secondary messenger ppGpp interferes with cAMP-CRP regulon by promoting CRP acetylation in Escherichia coli
Source: PLoS One. 2021 Oct 27;16(10):e0259067. doi: 10.1371/journal.pone.0259067 (PMC8550359; doi:10.1371/journal.pone.0259067)
Supplement: S1 File — (PDF) [file pone.0259067.s010.pdf]

Western blots from S3 Figure

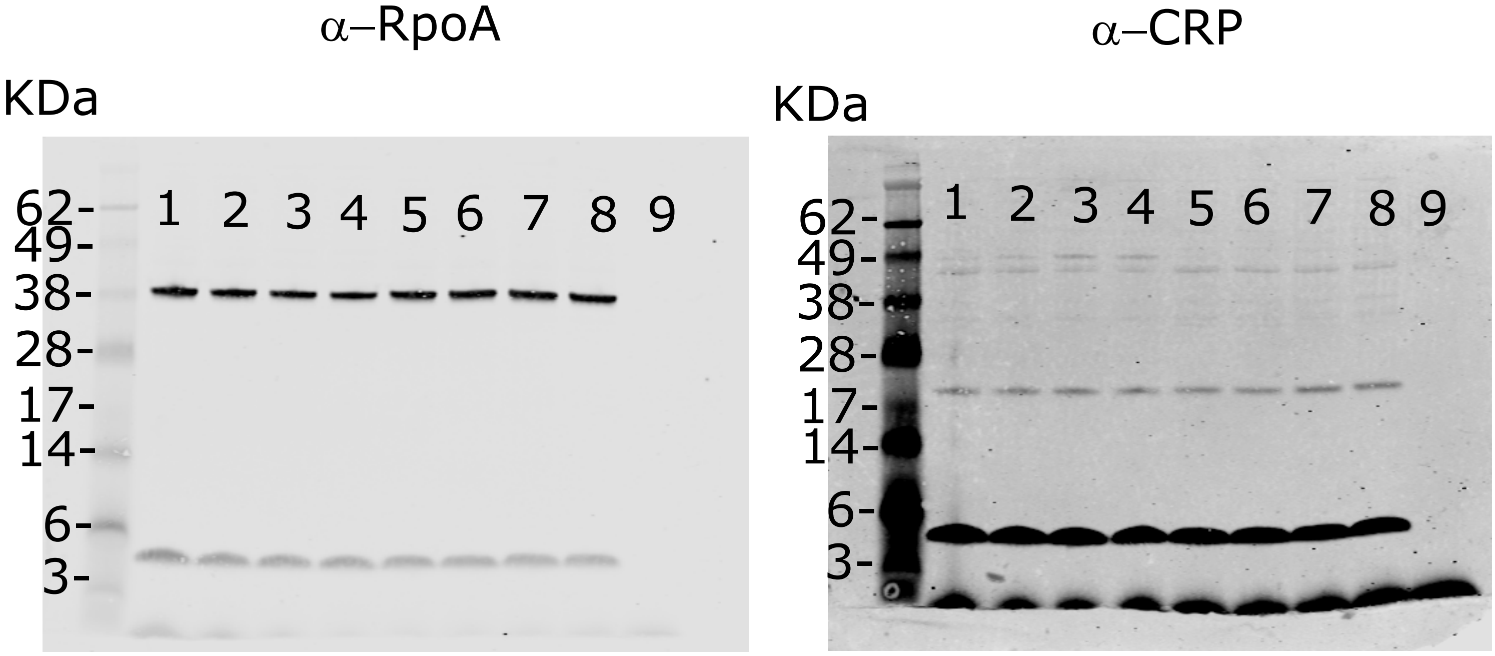


MWM: SeeBlue® Plus2 Pre-Stained Standard.

1 and 2 - MG1655 (WT) in M9 with glucose
3 and 4 - MG1655 (WT) in M9 with glycerol
5 and 6 - CF18005 (Δ*relA*) in M9 with glucose
7 and 8 - CF18005 (Δ*relA*) in M9 with glycerol
9 - Sample buffer (empty)

Image captured with Image Studio from Li-Cor

Western blots from S6 Figure

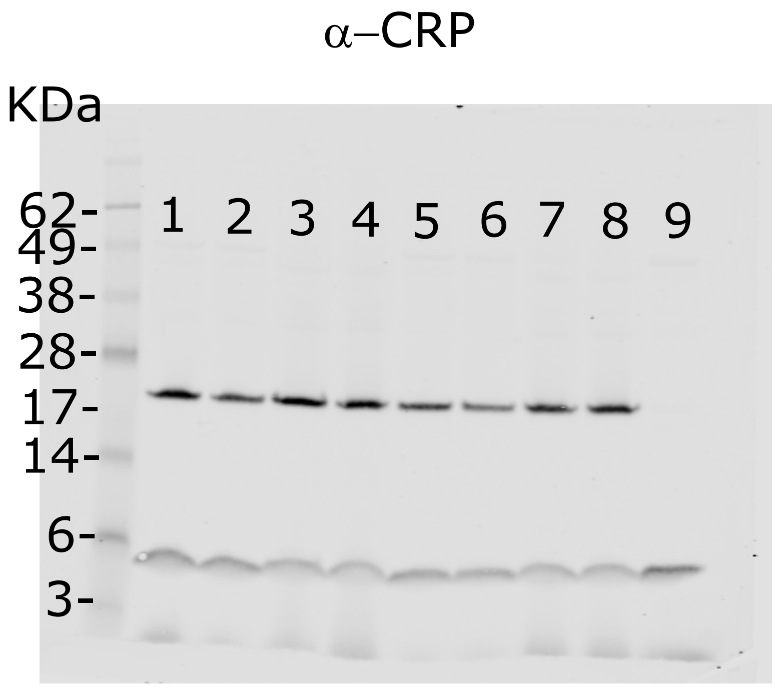


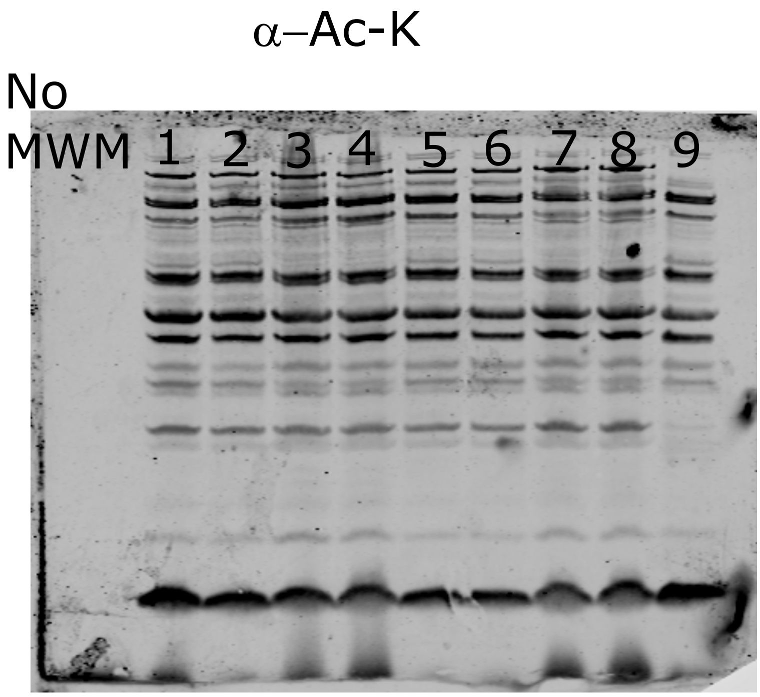


MWM: SeeBlue® Plus2 Pre-Stained Standard.

No Molecular Weight Marker was transfered in Ac-K blot

1 and 2 - MG1655 (WT) in M9 with glycerol
3 and 4 - CF18005 (Δ*relA*) in M9 with glycerol
5 and 6 - CF18566 (Δ*cobB*) in M9 with glycerol
7 and 8 - CF18582 (Δ*relA* Δ*cobB*) in M9 with glycerol
9 - CF6271 (Δ*crp*) in M9 with glucose

Image captured with Image Studio from Li-Cor

Western blots from S7 Figure

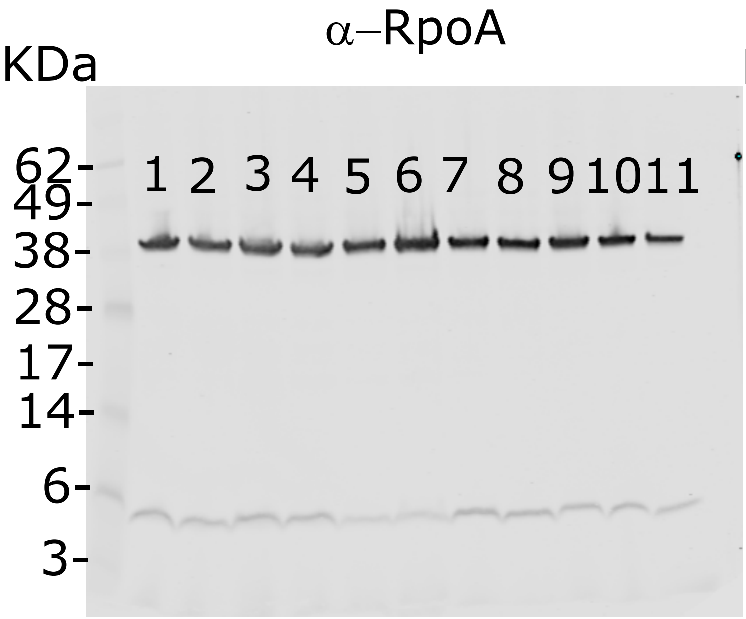


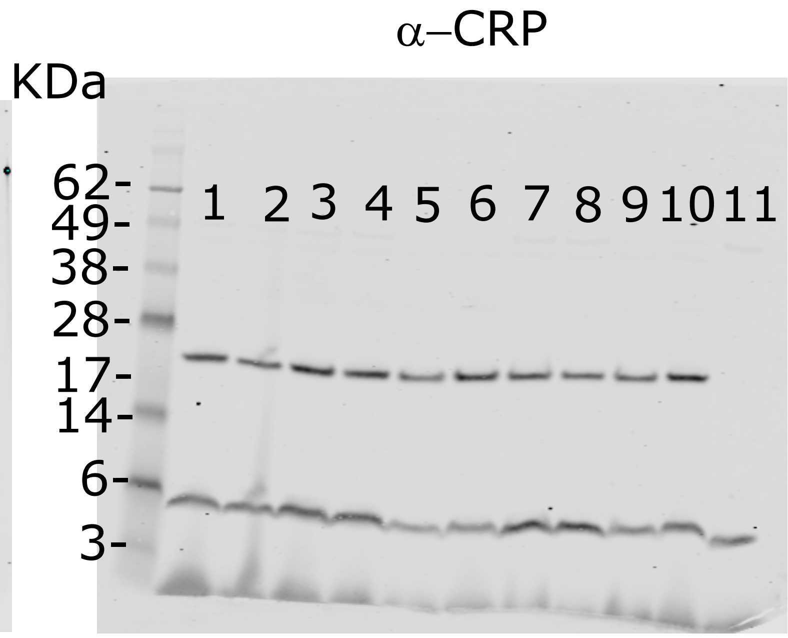


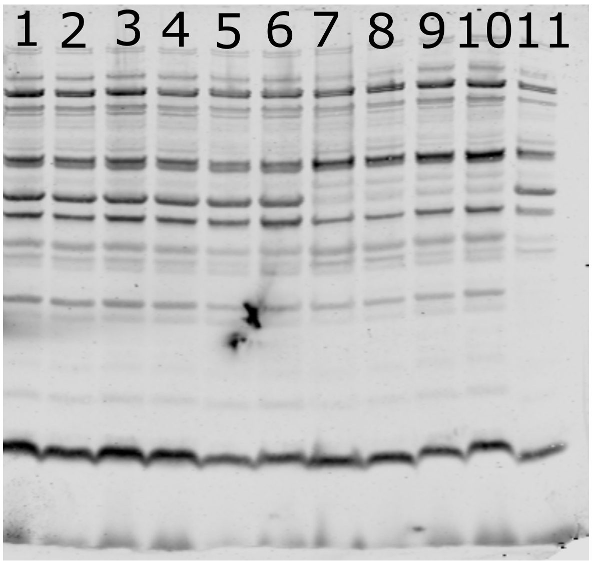


α–Ac-K

MWM: SeeBlue® Plus2 Pre-Stained Standard.

No Molecular Weight Marker was imaged in Ac-K blot

1 and 2 - MG1655 (WT) in M9 with glycerol

3 and 4 - CF18565 (Δ*ackA-pta*) in M9 with glycerol

5 and 6 - CF18531 (Δ*relA* Δ*ackA-pta*) in M9 with glycerol

7 and 8 - CF18572 (Δ*yfiQ*) in M9 with glycerol

9 and 10 - LFC1501 (Δ*relA* Δ*yfiQ*) in M9 with glycerol

11 - CF6271 (Δ*crp*) in M9 with glucose

Image captured with Image Studio from Li-Cor
